# Long-term effectiveness of mailed nicotine replacement therapy: study protocol of a randomized controlled trial 5-year follow-up

**DOI:** 10.1186/s12889-017-4586-z

**Published:** 2017-07-18

**Authors:** Vladyslav Kushnir, Peter Selby, Laurie Zawertailo, Rachel F. Tyndale, Scott T. Leatherdale, John A. Cunningham

**Affiliations:** 1 Centre for Addiction and Mental Health, 33 Russell St, Toronto, ON M5S 2S1 Canada; 2 Department of Pharmaceutical Sciences, University of Toronto, Toronto, M5S 3M2 Canada; 3 Department of Psychiatry, University of Toronto, Toronto, M5T 1R8 Canada; 4 Dalla Lana School of Public Health, University of Toronto, Toronto, M5T 3M7 Canada; 5 Department of Family and Community Medicine, University of Toronto, Toronto, M5G 1V7 Canada; 6 Department of Pharmacology and Toxicology, University of Toronto, Toronto, M5S 1A8 Canada; 7 School of Public Health and Health Systems, University of Waterloo, Waterloo, N2L 3G1 Canada; 8 Research School of Population Health, the Australian National University, Canberra, 2601 Australia

**Keywords:** Smoking, Tobacco, Nicotine dependence, Smoking cessation, Nicotine replacement therapy, Nicotine patches, Free distribution

## Abstract

**Background:**

Our group recently completed a randomized controlled trial, evaluating the efficacy of providing 5 weeks of free nicotine replacement therapy (NRT; in the form of the nicotine patch) by expedited postal mail without behavioral assistance to regular adult smokers interested in receiving it. The findings revealed that mailed provision of nicotine patches resulted in more than a doubling of quit rates at a six-month follow-up compared to a no intervention control group. While this trial provided evidence for the effectiveness of mailed nicotine patches in promoting cessation, the findings speak only to the short term effectiveness of this approach. As relapse to smoking is known to occur beyond the 6 month period, it is important to evaluate whether the net benefit of NRT in naturalistic settings can be maintained long-term. The present study aims to perform a 5-year follow-up survey of participants in the original trial to evaluate the long-term effectiveness of mailed NRT.

**Methods/Design:**

Trained interviewers will contact participants in the randomized controlled trial 5 years post-enrollment. A total of 924 participants will be eligible to be contacted. Interviewers will first assess participants’ smoking status and their level of nicotine dependence. Participants reporting not currently smoking will be asked whether they have smoked tobacco, even a puff, in the last 30 days (primary outcome measure: 30-day point prevalence abstinence), past 6 months (secondary outcome measure: prolonged 6-month abstinence), and since the 8-week follow-up survey (secondary outcome measure: > 4 year continuous abstinence). Interviewers will be blind to experimental condition at the time the primary outcome measure will be assessed. It is hypothesized that participants who received nicotine patches at baseline will display significantly higher quit rates at the 5-year follow-up as compared to participants who did not receive nicotine patches at baseline.

**Discussion:**

If the study finds that the mailed distribution of free NRT is effective at promoting long-term cessation, it would provide further evidence to move forward with policies designed to make NRT treatment readily and freely available to smokers who request it.

**Trial registration:**

ClinicalTrials.gov: NCT01429129, Registered 2 September 2011; NCT03097445, Registered 25 March 2017.

## Background

Smoking is a leading cause of preventable death worldwide. In Canada, smoking accounts for approximately 30% of all cancer deaths and is a risk factor for at least 18 types of cancer [1]. With over 2 million acute care hospital days (10.3% of all acute care hospital days) attributable to smoking in 2002, and related total costs exceeding $2.5 billion [2], tobacco smoking places significant social and economic burdens on Canadian society. The 2015 Canadian Tobacco, Alcohol and Drugs Survey reported that 13% of the population were current smokers, and while this represented a marked decline from 25% in 1999, it still accounts for a sizable 3.9 million Canadians [3].

To promote and support cessation among smokers, a number of medication options are available, with nicotine replacement therapy (NRT) recommended by many clinical practice guidelines as first-line treatment for those seeking pharmacological help [4–6]. Indeed, an extensive research base has demonstrated the efficacy of NRT in clinical settings, effectively increasing the rate of quitting smoking by 50–70%, irrespective of the clinical setting in which the smoker is treated [7]. In “real-world” settings however, where NRT is typically purchased over-the-counter and used without any behavioral support, cross-sectional and prospective cohort studies have failed to demonstrate that NRT use results in greater odds of cessation [8–10]. While this contradictory evidence challenges the relative effectiveness of over the counter availability and purchase of NRT in combatting smoking prevalence, our ability to make causal statements on the impact of NRT from population survey data alone is limited. This is because participants in those studies were not randomized and certain factors could have been systematically different between those who used NRT and those who did not.

With cost and access to interventions remaining significant barriers among most smokers who want to quit [11, 12], some jurisdictions have adopted the strategy of distributing free NRT in an effort to further reduce smoking prevalence. The distribution of free NRT by postal mail is ongoing in several countries, including the US [13–15] and Canada [16, 17]. In Ontario, the mass distribution of free NRT has been ongoing since the beginning of 2006, with the Ontario Ministry of Health and Long-Term Care devoting over $14 million (approximately $235 per participating smoker) to this endeavor [18]. Through the Smoking Treatment of Ontario Patients (STOP) Study, over 13,000 smokers have received the free NRT (primarily in the form of Nicotine Patches) via the mail, and while this phase of the distribution program has recently concluded, NRT continues to be offered for free combined with counseling through community and regional healthcare organizations [17]. The mass distribution of NRT has been also undertaken in British Columbia and proposals are underway that it be tried in other provinces as well. While commonly implemented as part of smoker’s helplines, the distribution of free NRT with minimal behavioral over-the-phone support has also demonstrated promising results in this intervention increasing short-term cessation rates among large populations of smokers [13, 14, 19–21]. However, because the evaluation of such mass distribution initiatives to-date has been primarily restricted to pre-post assessment without a randomly assigned control group [13, 16], there has been no reliable information on the efficacy of the approach. In addition to the absence of strong evidence on the effectiveness of NRT in ‘real-world’ settings where there is no additional behavioral support, this pointed to the need for a randomized controlled trial (RCT) on the effectiveness of mass distribution of free NRT in settings similar to how it is likely to be employed by the majority of people using it to try to quit smoking.

### Randomized controlled trial of mailed free NRT

Addressing the need for strong evidence on the effectiveness of NRT in naturalistic settings, we have recently completed an RCT evaluating the efficacy of providing 5 weeks of free NRT (in the form of the nicotine patch) by expedited postal mail without behavioral assistance [22, 23]. Employing random digit dialing of home and cellular telephone numbers to identify regular adult smokers across Canada interested in participating in a longitudinal survey, the trial recruited individuals hypothetically interested in receiving nicotine patches if offered for free, would use them within one week of receipt and those who endorsed no contraindications for nicotine patch use (*n* = 999). Participants interested in free nicotine patches and deemed eligible to participate were randomly assigned either to receive a 5-week course of nicotine patches by expedited postal mail or to a no-intervention control group. The 5-week course of nicotine patches was chosen because it mimicked the quantity of nicotine patches sent in the Ontario-based mass distribution initiative and is in line with the amount of nicotine patches mailed in other mass distribution initiatives [13, 17]. Participants randomized to the control group were not offered the nicotine patches or any other intervention and, most importantly, were unaware that the nicotine patches were being offered to others, thus allowing for a strong test of the hypothesis.

Follow-up rates were 86.4% at 8-weeks and 80.5% at 6-months. The findings revealed that the provision of free nicotine patches via mail resulted in more than a doubling of 30-day abstinence quit rates at a six-month follow-up compared to the no-intervention control group (Intent to treat: 7.6% versus 3.0%; odds ratio of 2.65, *p* = .002; Complete case: 9.8% versus 3.6%; odds ratio 2.89, *p* = .001). The results of this trial have twofold importance. First, they provided strong evidence of the effectiveness of only 5 weeks of nicotine patches as a tobacco cessation aid in in real-world settings, and second, they underlined the worth of mass distribution of free NRT initiatives in promoting tobacco cessation on a population level.

### Rationale and aims of this study

While the original trial provided evidence for the effectiveness of nicotine patches as a tobacco cessation aid in real-world settings, the findings speak only to the short-term effectiveness of NRT. As evidence for NRT effectiveness has been largely restricted to a final follow-up of 6–12 months after the start of treatment [24, 25], and relapse to smoking is known to occur beyond this period [26], it is important to evaluate whether the net benefit of NRT in naturalistic settings can be maintained long-term. Therefore, this study is a single follow-up survey of participants from the original randomized controlled trial, 5-years post enrollment, evaluating the effectiveness of mailed distribution of free NRT in promoting and maintaining long-term smoking cessation. The primary hypothesis is that participants who received nicotine patches at baseline will display significantly higher quit rates at the 5-year follow-up as compared to participants who did not receive nicotine patches at baseline.

## Methods

### Follow-up survey procedures

The 5-year follow-up surveys will be conducted by trained interviewers at the Survey Research Centre (SRC) of the University of Waterloo. The SRC had conducted recruitment and surveys as part of the original RCT, and as such, is appropriate to maintain consistency between follow-up periods. Interviewers will initially attempt to contact all eligible participants using the telephone numbers on record from the original trial. A total of 924 participants of the 999 initially recruited (92.5%) will be eligible to be contacted as part of the study (24 refused to be recontacted for a long-term follow-up; 47 withdrew from the trial; 4 deceased). Similar to the methodology used to conduct 6-month follow-up surveys in the original trial, the SRC will employ a rigorous call schedule of 30 call attempts per record, making up to 2 call attempts per day, with the majority of call placements made during the evening and weekend periods.

It is reasonable to expect however, that despite efforts by the SRC to contact individuals using their phone numbers on record, a proportion of phone numbers will no longer be correct or in service, likely due to participants’ relocation or changes in telephone service providers over the 5-year period since enrollment in the trial. A comprehensive Internet search strategy of publicly available sources and phone directories (i.e., www.canada411.ca, https://411.ca/person, and Google’s custom search function through www.locatefamily.com) will thus be undertaken by a trained research analyst to locate the contact information and interview participants who could not be initially reached using the phone numbers on record.

Once located and contacted, participants will be informed of the importance of completing the follow-up survey, its relatively short duration of 10 to 15 min, and a $30 remuneration honorarium for its completion. Upon completion of the survey, an up-to-date address and other contact information will be gathered and a $30 honorarium cheque will be subsequently mailed.

The limited research on the long-term (>4 years) effectiveness of smoking cessation interventions has documented that despite no contact with participants for over 3 years, follow-up rates as high as 85% can be achieved by means of mailed questionnaires and telephone calls [27], and even higher when recontacting only those who were abstinent at an earlier follow-up [28–30]. There can thus be reasonable confidence that using the proposed intensive investigative techniques a follow-up rate of greater than 63% will be obtained, as necessary for evaluating outcomes with adequate power in this study.

### Ethical approval

The research methods to be used in this study have been approved by the standing ethics review committee of the Centre for Addiction and Mental Health (CAMH).

### Content of 5-year post-intervention follow-up survey:

At the onset of the follow-up survey participants will be informed that the long-term follow-up survey seeks to examine their smoking behavior over the past 5 years. Interviewers will be masked to participants’ experimental condition. Initially, interviewers will assess participants’ current smoking status by evaluating the number of cigarettes smoked per day, and classes of smoking abstinence (detailed below) if zero cigarettes smoked per day are endorsed. In addition, participants who endorse abstinence will be asked when they quit smoking, the number of times they made serious quit attempts over the past 5 years, the duration of their longest quit, and whether they have purchased NRT, prescribed medication and/or received any behavioral or pharmacological support in their quit efforts.

These questions will specifically explore the types of cessation intervention tools that were used over the past 5 years, including electronic cigarettes, self-help/online methods, as well as participation and/or receipt of various forms of advice and counselling from healthcare professionals or smoking cessation specialists. As some individuals are known to initiate use of other tobacco products after stopping use of conventional cigarettes, such as cigars or non-combustible products such as smokeless tobacco or electronic cigarettes, participants who report smoking abstinence will be asked if they currently use other tobacco or nicotine containing products. This will allow us to examine whether people who quit cigarettes compensate with other forms of nicotine delivery.

Participants reporting currently smoking cigarettes will be evaluated on their level of nicotine dependence and asked of their motivation to quit. These individuals will also be asked how many times since the baseline survey (in the past 5 years) they have stopped smoking for even a day because they were trying to quit, the longest duration of a quit attempt, and what smoking cessation resources, including NRT, they had purchased or used in their quit attempts. Identical questions will apply to individuals who are identified as continuous smokers at each follow-up throughout the 5-year duration of the study, as well as to those who were abstinent at the 6-month follow-up and have now relapsed. However, the latter will be further asked to provide the duration of abstinence since quitting in the original trial.

As smoking impacts on health-related quality of life (HRQol) [31], the HRQol will be additionally measured in all responders using the World Health Organization Quality of Life Instrument (WHOQOL-BREF) [32]. Further, participants will be asked to identify any smoking-related adverse health diagnoses over the past 5 years for which there is sufficient evidence to infer a causal link [33], as well as report on past 12-month diagnoses of any psychiatric disorders.

### Measures

The following measures and tools will be employed:

#### Smoking abstinence

If a participant endorses smoking zero cigarettes per day, three classes of abstinence will be measured (point prevalence, prolonged, and continuous), as each measure characterizes former smokers from a different perspective – and hence effectiveness of mailed free nicotine patches – and has different strengths and weaknesses [34, 35]. A 7-day and 30-day *point prevalence abstinence* will be initially assessed, defined as not smoking, even a puff, for the past 7 days or 30 days, respectively. This measure of abstinence is particularly advantageous for capturing the dynamic process of change in natural environments such as in the present trial because when measured at prolonged periods after intervention, it takes into account smokers’ delayed initiation and action to quit. While point prevalence rates allow for lapses and relapses to occur and do not necessarily categorize these instances as treatment failures, point prevalence rates are disadvantaged by capturing only the more immediate health benefits of quitting. Nevertheless, *the 30-day point prevalence abstinence at 5 years is the primary outcome measure primarily because it is consistent with the primary outcome measure used in the original trial.* Participants reporting 30-day point prevalence abstinence will be further evaluated for *prolonged 6-month abstinence;* that is, not smoking even a puff in the past 6 months. A measure of more stable change than the point-prevalence abstinence, the 6-month prolonged abstinence effectively evaluates individuals in the maintenance stage of the transtheoretical model of behavioral change [36], where many of the long-term health benefits of quitting smoking begin to emerge [37]. Subsequently, the long-term impact of free nicotine patch provision will be assessed using the *continuous abstinence* measure – not smoking even a puff since the 8-week follow-up survey (>4 year continuous abstinence). Although continuous abstinence typically refers to abstinence that begins on the quit date or start of intervention until the last assessment time point, the exact start date of nicotine patch use among experimental group participants of the original trial is not known, because the nicotine patches were mailed to participants. Therefore, evaluating continuous abstinence from the expected end of treatment for nicotine patch recipients (8-week follow-up survey) would reflect not smoking since the intervention and would assess the stability of abstinence over time most accurately. While this measure of abstinence would be most appropriate in attributing the role of mailed free nicotine patch provision to the reduction of smoking-attributed morbidity, most smokers in naturalistic settings such as in the current trial do not quit without experiencing lapses or relapses [34, 35], thus this measure of abstinence would be most conservative and result in reduced rates.

#### Nicotine dependence

Nicotine dependence will be assessed using the Fagerström Test for Nicotine Dependence (FTND) [38], a 6-item, well validated, highly reliable and most frequently used measure of nicotine dependence severity that closely matches biochemical indices of smoking heaviness. [39, 40].

#### Motivation to quit

For respondents currently smoking, motivation to quit smoking will be assessed using the Transtheoretical Model of Change framework, which conceptualizes motivation as readiness for change and measures motivation as categorical stages of change [41]. This theory conceptualizes motivation sequentially, categorizing motivation into five stages of change: precontemplation, contemplation, preparation, action and maintenance. Individuals in the *precontemplation stage* endorse an intent to quit but not in the next 6 months, those in the *contemplation stage* plan to quit in the next 6 months but not in the next 30 days, the *preparation stage* is defined as a plan to quit in the next 30 days, individuals in the *action stage* are those who are actively trying to stop smoking or have quit in the past 6 months, and those in the *maintenance stage* are typically regarded as having quit smoking for 6 months or more. Within addictions research, this model of motivation for change has often been used to predict treatment success and understand the recovery process of addictions.

#### Health-related quality of life

The World Health Organization Quality of Life Instrument (WHOQOL-BREF) [32] will be used to assess participants’ well-being from four domains of quality of life: physical, psychological, social and environmental. This person-centered assessment tool has been demonstrated to perform well on tests of internal consistency, discriminant validity and construct validity, and is cross-culturally sensitive [32].

#### Smoking-attributed morbidity

All participants will be asked to report any smoking-related adverse health diagnoses over the past 5 years. Response options will present health problems for which there is sufficient evidence to infer a causal link between smoking and the respective health problem, as derived from the U.S. Department of Health and Human Services report on *The Health Consequences of Smoking—50 Years of Progress: A Report of the Surgeon General, 2014* [33].

### Outcome measures

#### Primary outcome

Consistent with primary outcomes of the original trial and the overwhelming majority of mass distribution of NRT studies, the primary outcome measure will be self-reported 30-day point prevalence abstinence, at 5-years post-intervention. While biochemical validation would increase confidence in the results, short-term outcomes from the trial (8-week and 6-month follow-ups) have demonstrated that collecting saliva samples via postal mail is not feasible because many participants will not return their samples, and those returned are often evaporated [23]. However, there is sufficient evidence of the reliability of self-reported tobacco cessation without biochemical validation and self-report is the recommended approach for studies of this type [42–44].

#### Secondary outcomes

The secondary outcome measures will be self-reported prolonged 6-month abstinence and continuous abstinence since the 8-week follow-up survey (> 4 year abstinence). Empirical research has demonstrated that while recall of brief quit attempts followed by relapse to smoking is prone to error [45], among respondents whose quit attempt is ultimately successful their ability to place their smoking cessation in time is actually highly accurate [46]. Additional secondary outcomes will be HRQol, exploring tobacco cessation-mediated differences in health-related quality of life between experimental and control conditions, as well as smoking-related morbidity over the past 5 years.

### Data analysis

#### Power analysis

Previous research on the long-term effectiveness of NRT has indicated that a 30% relapse rate can be reasonably expected at follow-up times beyond 12 months from the start of treatment, and is relatively stable thereafter [47]. Based on this relapse rate and the abstinence rates of completers at 6 months (9.8% of smokers in the experimental group and 3.6% of those in the control group), the quit rates for the experimental and control conditions in the present study can be estimated to be 6.86% and 2.52%, respectively at the 5-year follow-up. Under these assumptions, a power calculation was conducted by Monte Carlo simulation with 10,000 replications for each sample size, where the outcome (quit smoking/active smoker) at the end of the 5 years was considered to be a binomial variable with proportion parameters of 6.86% and 2.52% and equal sample sizes in each group. For each replication a logistic regression was adjusted with study group as the independent variable and the result was considered significant whenever the *p*-value for the experimental group reached the 0.05 threshold. The proportion of times differences in quit rates between study groups were significantly different across the 10,000 replications for a given sample size was the estimated power for that sample size. It was determined that a sample size of 290 subjects per group would be necessary to detect the expected 4.34% difference in quit rates with 80% power and 0.05 confidence level. While a 4.34% difference in quit rates might seem small when compared to the results of a controlled clinical trial, it still represents a 172% decrease in smoking rates and is substantial from a public health perspective. Overall, a total of 580 of 924 participants (62.8%) will need to be followed up at the 5-years in order to have adequate power for a complete case analysis. Using the planned investigative techniques to contact participants who may have moved or changed telephone numbers, we have confidence that this follow-up rate can be achieved or exceeded.

An alternative approach to calculating power and necessary sample size to observe the estimated quit rates at 5 years utilizes the assumption that it is highly likely that the proportion of quitters in each group at 5 years will be similar to what was found at 6 months, and there is reasonable confidence that it will not be greater. Such information can be used to conduct a Bayesian logistic regression at the end of 5 years where the binary outcome (quit smoking/active smoker) is regressed on the dichotomous study group variable (experimental/control). The a priori distribution for the coefficient of the study group is defined as normally distributed with the mean equal to the coefficient of the logistic regression adjusted at 6 months and standard deviation 3 times larger than the standard deviation found at 6 months, which allows for a good level of uncertainty. To calculate the equivalent power for such model, Monte Carlo simulations were used. For both experimental and control groups a binomial data set was generated with sample size *n* and quit proportions 6.86% and 2.52%, respectively, to reflect expected quit rates at the 5 year follow-up. A Bayesian logistic regression was subsequently fit to this data, as defined above, producing a 95% posterior credibility interval for the model coefficient related to each study group. For each sample size, synthetic data was generated 1000 times and the proportion of time that the 95% posterior credibility interval did not include the value 0 (zero) was equivalent to the classical definition of power. Performing such simulations it was determined that a sample size of 260 subjects per group would yield 82% power to detect the expected differences in quit rates at 5 years, using the credibility intervals of 95%. These simulations were conducted with package rstan [48] and rstanarm [49] from R version 3.3.2 [50]. Data analyses at the conclusion of the study will also use the same software.

#### Analysis plan

Similar to outcomes evaluation in the original trial, all analysis will employ an intent-to-treat approach such that all subjects will remain in the group to which they were randomly assigned, regardless of treatment adherence. *Primary analyses* will employ a binary logistic regression to evaluate the odds of smoking cessation among nicotine patch recipients compared to the no intervention control group, 5-years post-baseline. These analyses will be initially conducted using a conservative approach where subjects with missing data due dropout or loss to follow-up are considered active smokers, followed by analyses using only the 5-year follow-up survey completers. Exploratory Bayesian logistic regression analyses will also be performed akin to the primary, classical analytical approach. Secondary analyses will use logistic regression analyses to examine the effect of free nicotine patch distribution on prolonged 6-month abstinence and continuous abstinence (>4 years). Further, multiple regression analyses will explore possible mediating effects of additional pharmacotherapeutic or behavioral support in the maintenance of smoking cessation between the 6 month and 5-year follow-up and multivariate regression analyses will be used to examine the association between cessation outcomes and demographic and baseline smoking characteristics (i.e., number of cigarettes smoked per day, level of nicotine dependence, motivation to quit, etc.,). Similar multivariate analyses will also be conducted to evaluate the role of these variables in predicting relapse between 6-month and 5-year follow-ups. These analyses will be further complemented with log rank survival analyses to determine whether relapse rates post-8 weeks and 6 months are different between experimental and control conditions. Separate linear regression analysis will also be employed to test the impact of receiving NRT on Health Related Quality of Life (using the WHOQOL-BREF composite score as the dependent measure). It is predicted that subjects in the experimental condition will display significantly improved HRQol in comparison to those in the control condition at the 5-year follow-up. This impact on HRQol will be mediated by compliance with the NRT use protocol and success at tobacco cessation. Finally, separate univariate analyses of variance will explore differences between groups in the number of individual and total smoking-attributed health diagnoses over the past 5 years.

## Discussion

Through the Canadian National Tobacco Control Strategy, the federal, provincial and territorial ministries of health have committed to reduce the number of tobacco-related deaths and illnesses over a 10-year period [51]. In order to attain this goal, it is essential to evaluate new means of promoting tobacco cessation and further evaluate the net long-term benefit of such interventions. The most effective way to reduce the risk of cancer is to quit smoking, which motivates the proposed research to investigate: 1) the long-term effectiveness of NRT as a smoking cessation aid in real-world settings; and 2) whether the provision of free NRT to Canadian smokers is an effective population-level strategy to reduce the prevalence of smoking and, consequently, the incidence of smoking-attributed morbidity. If the study finds that the mailed distribution of free NRT is effective at promoting long-term cessation, it would provide further evidence to move forward with policies designed to make NRT treatment readily and freely available to smokers who request it. Overall, the project will contribute in advancing evidence-based treatment of tobacco dependence, as well as provide data that will inform tobacco control guideline developers, funders of services, and consumers who smoke.

## Abbreviations

FTND: Fagerström Test for Nicotine Dependence
HRQol: Health-related quality of life
NRT: Nicotine replacement therapy
RCT: Randomized controlled trial
SRC: Survey research centre

## References

[1] Canadian Cancer Society/National Cancer Institute of Canada: Canadian Cancer Statistics 2005. Toronto, Canada, 2005. [http://www.cancer.ca/~/media/cancer.ca/CW/cancer%20information/cancer%20101/Canadian%20cancer%20statistics/Canadian-Cancer-Statistics-2005-EN.pdf?la=en]. Accessed 29 June 2009.

[2] Baliunas D, Patra J, Rehm J, Popova S, Taylor B (2007). Smoking-attributable morbidity: acute care hospital diagnoses and days of treatment in Canada, 2002. BMC Public Health.

[3] Canadian Tobacco Alcohol and Drugs Survey (CTADS): 2015 summary. [https://www.canada.ca/en/health-canada/services/canadian-tobacco-alcohol-drugs-survey/2015-summary.html]. Accessed June 21, 2017.

[4] West R, McNeill A, Raw M (2000). Smoking cessation guidelines for health professionals: an update. Health Education Authority Thorax.

[5] The 2008 PHS Guideline Update Panel. Treating tobacco use and dependence: 2008 update U.S. Public Health Service Clinical Practice Guideline executive summary. Respir Care. 2008;53:1217–22. 18807274

[6] Le Foll B, Melihan-Cheinin P, Rostoker G, Lagrue G (2005). Smoking cessation guidelines: evidence-based recommendations of the French health products safety agency. European Psychiatry.

[7] Stead LF, Perera R, Bullen C, Mant D, Hartmann-Boyce J, Cahill K, Lancaster T (2012). Nicotine replacement therapy for smoking cessation. The Cochrane database of systematic reviews.

[8] Alpert HR, Connolly GN, Biener L (2013). A prospective cohort study challenging the effectiveness of population-based medical intervention for smoking cessation. Tob Control.

[9] Kotz D, Brown J, West R (2013). 'Real-world' effectiveness of smoking cessation treatments: a population study. Addiction.

[10] Kotz D, Brown J, West R (2014). Prospective cohort study of the effectiveness of smoking cessation treatments used in the "real world". Mayo Clin Proc.

[11] Land T, Warner D, Paskowsky M, Cammaerts A, Wetherell L, Kaufmann R, Zhang L, Malarcher A, Pechacek T, Keithly L (2010). Medicaid coverage for tobacco dependence treatments in Massachusetts and associated decreases in smoking prevalence. PLoS One.

[12] Leatherdale ST, Shields M (2009). Smoking cessation: intentions, attempts and techniques. Health Rep.

[13] Cummings KM, Fix B, Celestino P, Carlin-Menter S, O'Connor R, Hyland A (2006). Reach, efficacy, and cost-effectiveness of free nicotine medication giveaway programs. Journal of Public Health Management Practice.

[14] Miller N, Frieden TR, Liu SY, Matte TD, Mostashari F, Deitcher DR, Cummings KM, Chang C, Bauer U, Bassett MT (2005). Effectiveness of a large-scale distribution programme of free nicotine patches: a prospective evaluation. Lancet.

[15] Davis KA, Coady MH, Mbamalu IG, Sacks R, Kilgore EA (2013). Lessons learned from the implementation of a time-limited, large-scale nicotine replacement therapy giveaway program in new york city. Health Promot Pract.

[16] Selby P, Zawertailo L, Dragonetti R, Bondy SJ, Ho J: Stop Smoking Therapy for Ontario Patients (Stop Study): Methods for Free NRT Distribution. In: *World Conference on Tobacco or Health:* 2006*; Washington, D.C.*; 2006.

[17] Zawertailo L, Dragonetti R, Bondy SJ, Victor JC, Selby P (2013). Reach and effectiveness of mailed nicotine replacement therapy for smokers: 6-month outcomes in a naturalistic exploratory study. Tob Control.

[18] Selby P, Zawertailo L, Dragonetti R. The STOP study. Ninth Interim Progress Report to the Ministry of Health Promotion; 2009.

[19] Bush TM, McAfee T, Deprey M, Mahoney L, Fellows JL, McClure J, Cushing C (2008). The impact of a free nicotine patch starter kit on quit rates in a state quit line. Nicotine Tob Res.

[20] Swartz SH, Cowan TM, Klayman JE, Welton MT, Leonard BA (2005). Use and effectiveness of tobacco telephone counseling and nicotine therapy in Maine. Am J Prev Med.

[21] Tinkelman D, Wilson SM, Willett J, Sweeney CT (2007). Offering free NRT through a tobacco quitline: impact on utilisation and quit rates. Tob Control.

[22] Cunningham J, Leatherdale S, Selby P, Tyndale R, Zawertailo L, Kushnir V (2011). Randomized controlled trial of mailed nicotine replacement therapy to Canadian smokers: study protocol. BMC Public Health.

[23] Cunningham JA, Kushnir V, Selby P, Tyndale RF, Zawertailo L, Leatherdale ST (2016). Effect of mailing nicotine patches on tobacco cessation among adult smokers : a randomized clinical trial. JAMA Intern Med.

[24] Cahill K, Stevens S, Perera R, Lancaster T (2013). Pharmacological interventions for smoking cessation: an overview and network meta-analysis. Cochrane Database Syst Rev.

[25] Stead LF, Koilpillai P, Fanshawe TR, Lancaster T (2016). Combined pharmacotherapy and behavioural interventions for smoking cessation. Cochrane Database Syst Rev.

[26] Hughes JR, Peters EN, Naud S (2008). Relapse to smoking after 1 year of abstinence: a meta-analysis. Addict Behav.

[27] Nohlert E, Ohrvik J, Tegelberg A, Tillgren P, Helgason AR (2013). Long-term follow-up of a high- and a low-intensity smoking cessation intervention in a dental setting--a randomized trial. BMC Public Health.

[28] Blondal T, Gudmundsson LJ, Olafsdottir I, Gustavsson G, Westin A (1999). Nicotine nasal spray with nicotine patch for smoking cessation: randomised trial with six year follow up. BMJ.

[29] Glavas D, Rumboldt M, Rumboldt Z (2003). Smoking cessation with nicotine replacement therapy among health care workers: randomized double-blind study. Croat Med J.

[30] Clavel-Chapelon F, Paoletti C, Benhamou S (1997). Smoking cessation rates 4 years after treatment by nicotine gum and acupuncture. Prev Med.

[31] Sales MP, Oliveira MI, Mattos IM, Viana CM, Pereira ED (2009). The impact of smoking cessation on patient quality of life. Brazilian Journal of Pulmonology.

[32] Skevington SM, Lotfy M, O'Connell KA, Group W (2004). The World Health Organization's WHOQOL-BREF quality of life assessment: psychometric properties and results of the international field trial. A report from the WHOQOL group. Qual Life Res.

[33] U.S. Department of Health and Human Services. The Health Consequences of Smoking - 50 years of Progress: A Report of the Surgeon General. In. Atlanta, GA: U.S. Department of Health and Human Services, Centers for Disease Control and Prevention, National Center for Chronic Disease Prevention and Health Promotion, Office on Smoking and Health; 2014.

[34] Velicer WF, Prochaska JO, Rossi JS, Snow MG (1992). Assessing outcome in smoking cessation studies. Psychol Bull.

[35] Velicer WF, Prochaska JO (2004). A comparison of four self-report smoking cessation outcome measures. Addict Behav.

[36] Prochaska JO, Velicer WF (1997). The transtheoretical model of health behavior change. Am J Health Promot.

[37] U.S. Department of Health and Human Services. The Health Benefits of Smoking Cessation: A Report of the Surgeon General. In. Atlanta: U.S. : Department of Health and Human Services, Centers for Disease Control and Prevention, Center for Chronic Disease Prevention and Health Promotion, Office on Smoking and Health; 1990.

[38] Heatherton TF, Kozlowski LT, Frecker RC, Fagerstrom KO (1991). The Fagerstrom test for nicotine dependence: a revision of the Fagerstrom tolerance questionnaire. Br J Addict.

[39] Pomerleau CS, Carton SM, Lutzke ML, Flessland KA, Pomerleau OF (1994). Reliability of the Fagerstrom tolerance questionnaire and the Fagerstrom test for nicotine dependence. Addict Behav.

[40] Van Overmeire IP, De Smedt T, Dendale P, Nackaerts K, Vanacker H, Vanoeteren JF, Van Laethem DM, Van Loco J, De Cremer KA. Nicotine Dependence and Urinary Nicotine, Cotinine and Hydroxycotinine Levels in Daily Smokers. Nicotine Tob Res. 2016;18:1813–9. 10.1093/ntr/ntw099 27083213

[41] Prochaska JO, DiClemenete CC, Norcross JC (1992). In search of how people change: applications to addictive behaviours. Am Psychol.

[42] Wong SL, Shields M, Leatherdale S, Malaison E, Hammond D (2012). Assessment of validity of self-reported smoking status. Health Rep.

[43] Patrick DL (1994). Toward an epidemiology of disablement. Am J Public Health.

[44] West R, Zatonski W, Przewozniak K, Jarvis MJ (2007). Can we trust national smoking prevalence figures? Discrepancies between biochemically assessed and self-reported smoking rates in three countries. Cancer Epidemiol Biomark Prev.

[45] Hughes JR, Solomon LJ, Naud S, Fingar JR, Helzer JE, Callas PW (2014). Natural history of attempts to stop smoking. Nicotine Tob Res.

[46] US Centers for Disease Control/National Center for Health Statistics: Cognitive Research on Response Error in Survey Questions on Smoking: DAINE Publishing; 1992.

[47] Etter JF, Stapleton JA (2006). Nicotine replacement therapy for long-term smoking cessation: a meta-analysis. Tob Control.

[48] Stan Development Team. rstanarm: Bayesian applied regression modeling via Stan. version:2.13.1; 2016.

[49] Stan Development Team. RStan: the R interface to Stan. version:2.14.1; 2016.

[50] R Core Team: R: A language and environment for statistical computing. R Foundation for Statistical Computing. Vienna, Austria; 2016.

[51] Tobacco Control Liaison Committee of the Pan-Canadian Public Health Network. The National Strategy: Moving Forward - The 2006 Progress report on tobacco control. In. Ottawa: Health Canada; 2006.

